# Therapeutic Potential of Gene-Modified Regulatory T Cells: From Bench to Bedside

**DOI:** 10.3389/fimmu.2018.00303

**Published:** 2018-02-16

**Authors:** Wook-Jin Chae, Alfred L. M. Bothwell

**Affiliations:** ^1^Department of Immunobiology, Yale University School of Medicine, New Haven, CT, United States

**Keywords:** regulatory T cell, autoimmunity, cancer, gene modification, therapy

## Abstract

Regulatory T cells (Tregs) are an important subset of adaptive immune cells and control immune reactions for maintaining homeostasis. Tregs are generated upon their encounter with self or non-self-antigen and mediate tolerance or suppress aberrant immune responses. A high level of specificity of Tregs to recognize antigen(s) suggested their instrumental potential to treat various inflammatory diseases. This review will first introduce seminal basic research findings in the field of Tregs over the last two decades pertinent to therapeutic approaches in progress. We will then discuss the previous approaches to use Tregs for therapeutic purposes and the more recent development of gene-modification approaches. The suppressive function of Tregs has been studied intensively in clinical settings, including cancer, autoimmunity, and allotransplantation. In cancer, Tregs are often aberrantly increased in their number, and their suppressor function inhibits mounting of effective antitumor immune responses. We will examine potential approaches of using gene-modified Tregs to treat cancer. In autoimmunity and allotransplantation, chronic inflammation due to inherent genetic defects in the immune system or mismatch between organ donor and recipient results in dysfunction of Tregs, leading to inflammatory diseases or rejection, respectively. Since the recognition of antigen is a central part in Treg function and their therapeutic use, the modulation of T cell receptor specificity will be discussed. Finally, we will focus on future novel strategies employing the therapeutic potential of Tregs using gene modification to broaden our perspective.

## Introduction

Regulatory T cells (Tregs) are an important T cell subpopulation that maintains immunological homeostasis. In the early 1970s, a few papers provided evidence for an inhibitory role of thymus-derived T cells. Seminal research by Gershon and Kondo introduced the concept of infectious tolerance (1, 2). Over the next decade, these thymus-derived T cells were termed “suppressor T cells.” Later, suppressor T cells were renamed as “regulatory T cells,” based on their capability to regulate autoimmunity. Since the 1990s, Tregs have been one of the most intense research fields in immunology. The negative regulation of immune responses by Tregs is vital in autoimmune and auto-inflammatory disorders, acute and chronic infection, allergy, metabolic inflammation, transplantation, and cancer. Accordingly, modulation of Tregs holds the therapeutic potential to treat numerous disease classes. The unique history of Tregs has been well-reviewed previously (3).

### Identifying Treg Markers and Mechanistic Studies of Treg Function in Mice

Initial attempts to isolate and to identify Tregs for immunological studies used IL-2 receptor α-chain (CD25), which is highly expressed on Tregs. Adoptive transfer of CD25^+^ Tregs prevented various experimental mouse models of autoimmunity (4–6). Other Treg markers were subsequently identified including lymphocyte activation gene (LAG)-3, CTLA-4, folate receptor-4, latency-associated peptide, and IL-35 (7–10). Often these markers were also expressed in activated effector CD4 T cells, necessitating the identification of more definitive markers of Tregs.

In this regard, one of the most important findings in Treg biology was the discovery of Treg lineage transcriptional factor FOXP3. Mutations in the *FOXP3* gene were identified in Immune dysregulation, polyendocrinopathy, enteropathy, X-linked syndrome (IPEX) human patients. The murine counterpart of IPEX patients, *scurfy* mice, lacks functional FOXP3 and showed similar phenotypes to IPEX patients (11–13). Two studies demonstrated the importance of FOXP3 in Treg development and function (14, 15). Development of biological tools including Red Fluorescent Protein (RFP) FOXP3 reporter mice, *Foxp3*-Diphtheria Toxin Receptor mice which permitted Treg depletion, and the development of Treg-specific *Foxp3^YFP–Cre^* mice which allowed the conditional deletion of a gene in Tregs, facilitated the understanding of Treg biology in mice (16–19).

CD4^+^FOXP3^+^ Tregs can be induced from peripheral CD4^+^ naïve T cells in the periphery by many factors such as tolerogenic dendritic cells expressing indoleamine 2,3-dioxygenase (IDO), commensal bacteria, retinoic acid, or transforming growth factor (TGF)-β and are designated peripheral Tregs (pTregs) to distinguish them from the thymic-derived Tregs (tTregs) (20–24). Similar to their tTregs, pTregs regulate immune responses in various types of inflammatory disease environments including spontaneous intestinal tumorigenesis, inflammatory bowel disease, asthma, and experimental autoimmune encephalomyelitis (EAE) (25–28). It has been reported that FOXP3^+^ Tregs express the immunosuppressive cytokine IL-10. Later, IL-10-expressing Tregs were further dissected into IL-10^+^FOXP3^+^ Tregs and Foxp3-negative type 1 Tregs (Tr1) that are induced by dexamethasone and Vitamin D (29–31).

Using genetic, biochemical, and molecular biological approaches, functional modules of Foxp3 such as dimerization/oligomerization of the transcriptional factor were identified, and the regulatory mechanism of Foxp3-mediated gene expression in Tregs was extensively studied (32–40). The molecular mechanism of stable FOXP3 expression has been under intense investigation by measuring DNA demethylation at the Treg-specific demethylated region (TSDR), a conserved CpG-rich region within the *Foxp3* locus where methylation maintains stable lineage commitment of Tregs (41, 42). In parallel to the regulation of FOXP3 expression, posttranslational modification by acetylation, ubiquitination, or phosphorylation has an important role in modulating the Foxp3-mediated transcriptional repression that is required for suppressor function (43–48).

### Human Tregs in Basic and Clinical Studies

In the past two decades, there has been significant progress in the understanding of regulatory mechanisms of tolerance in humans. Various markers for the identification of human Tregs were found including CD25, FOXP3, and CD127 (IL-7Rα chain) (49–52). Further studies revealed that human conventional T cells transiently express FOXP3 without acquiring suppressive activity (53). Human Tregs are functionally and phenotypically distinguished by their activation status. Suppressive Treg cells are CD45RA^+^FOXP3^lo^ in resting state and CD45RA*^−^*FOXP3^hi^ in activated state while CD45RA*^−^*FOXP3^lo^ T cells are non-suppressive. The proportion of the three subpopulations was markedly different between aged individuals, cord blood and patients with immunological diseases (54, 55).

Expansion of Tregs using rapamycin or induction of Tr1 cells has been utilized to induce polyclonal Tregs for clinical intervention (56, 57). Tr1 cells express similar markers to FOXP3^+^ Tregs such as CTLA-4, PD-1, CD39, and ICOS. Tr1 cells do not express FOXP3 constitutively, but they do express IL-10 and TGF-β once they are activated *via* T cell Receptor (TCR). Tr1 cells show bystander suppressor activity (58). IL-10 and TGF-β from Tregs inhibit effector CD4 T cells proliferation and production of effector cytokines, such as IL-2 and IFN-γ (59). Other than cytokine-mediated suppression, it is known that granzyme B-mediated cell death of myeloid APCs is mediated by the stable adhesion between HLA-class I molecules on Tr1 cells and CD112/CD115 on myeloid APCs (60).

In clinical settings, modification of TCR has been utilized to modulate Treg activity to intervene in various types of inflammatory diseases in an antigen-specific manner (61, 62). Treg-based therapies with freshly isolated or expanded Tregs have been implemented in clinical practice for patients undergoing allogeneic hemopoietic stem cell transplantation to prevent graft-versus-host disease (GVHD) (63), inhibiting rejection in solid organ transplantation and controlling autoimmunity in patients [e.g., Type 1 Diabetes (T1D)] (64). Since Tregs have multiple roles in a variety of clinical settings, the generation of gene-modified Tregs and administration of those Tregs *via* adoptive transfer is a promising approach to treat chronic inflammatory diseases, cancer, or rejection in transplantation medicine.

## Gene-Modified Tregs in Cancer Immunotherapy

Regulatory T cells are found at high frequencies in the tumor microenvironment in a variety of cancers (65). Analysis in a variety of human carcinomas suggested that the accumulation of Tregs in the tumor microenvironment is associated with a poor prognosis (65).

### Generating Tumor Antigen-Specific CAR^+^ Tregs

Over a decade ago, a seminal study proposed the therapeutic potential of genetically engineered T cells bearing a tumor antigen-specific TCR in cancer immunotherapy (66). Overexpression of the α and β chains of a specific TCR has been used as a traditional approach to engineer T cell specificity. The antigen-specific suppressor function of Tregs on effector T cells was demonstrated by the tumor antigen NY-ESO-1. Depletion of Tregs enabled the activation of NY-ESO-1-specific naïve CD4 T cells in healthy subjects and melanoma patients with NY-ESO-1-expressing tumors (67, 68). TCRs recognizing melanoma antigens have been successfully transduced in human Tregs *in vitro* (69). Interestingly, the affinity of the TCR did not affect the antigen-specific suppressive function. This indicated that tumor antigen-mediated TCR signals do not affect the function of fully differentiated Tregs *ex vivo*.

An alternative strategy is to transduce a chimeric antigen receptor (CAR) into Tregs to generate antigen-specific Tregs. CARs are synthetic proteins generated by fusing an extracellular domain for antigen recognition with transmembrane and signaling domains from the TCR and co-stimulatory receptors (70). The antigen-recognizing domain of a CAR is generated by a single-chain variable fragment (scFv) fusion protein of the complementarity determining regions of the heavy and light chains of a monoclonal antibody. A major advantage of generating scFvs is to avoid the limitation of MHC restriction. This expands the pool of treatable patients compared to the TCR overexpression approach. Expression and engineering of CARs that are specific to tumor antigens is now a primary interest in cancer immunotherapy employing CAR Tregs (71). Further studies for a more diverse set of tumor antigens are warranted to broaden the therapeutic potential of this approach.

### Modulating Foxp3 Expression in Tregs

Another approach to inhibit the suppressor function of Tregs is to downregulate FOXP3 expression. Use of lentiviral FOXP3 shRNA delivery inhibited Treg-like leukemia in mice (72). This lentiviral strategy was used to knockdown FOXP3 mRNA in human Tregs, and this approach demonstrated the loss of suppressor function, indicating that it has potential to be used in cancer immunotherapy (73). However, Tregs that are transduced with the lentivirus have not been tested for safety, and thus further research is needed. Stat3 has been reported to play a crucial role to maintain FOXP3 expression in human. Delivery of small interfering RNA (siRNA) for Stat3 into Tregs demonstrated the loss of the suppressor function (74). Recently, it has been reported that siRNA can be delivered in gold nanoparticles, circumventing the issue with a lentiviral system in human patients (75). A stable FOXP3 expression is dependent on posttranslational modification. Genetic or pharmacologic modulation of FOXP3 acetylation *via* the histone/protein acetyltransferases (HATs), p300, and CBP downregulated suppressive function of Treg and promoted antitumor immunity (76).

A recent study demonstrated that the pharmacologic inhibition of a single de-ubiquitination enzyme, Usp7, determines the fate of FOXP3 and Tip60 in Tregs, thus providing a target for therapeutic modulation of Treg function in antitumor immunity (77). It has been shown that selective small molecule inhibitors for the bromodomains of CREBBP/EP300 reduced FOXP3 expression, as well as expression of functional markers in Tregs (e.g., LAG-3, CTLA-4, and TIM-3) (78). It has also been reported that intranuclear interactomic inhibition of FOXP3 could abrogate suppressor function *via* nuclear delivery of FOXP3 (79). These approaches are promising at a preclinical stage, yet assessment of target-specific delivery of siRNA or Protein Transduction Domain-FOXP3 protein, and their side effects have not been assessed. Potential autoimmune responses should be considered when Treg dysfunction is implemented as a therapeutic approach in cancer immunotherapy. An additional concern is that a series of surprising reports found that a high incidence of tumor-infiltrating Tregs is associated with improved prognosis in cancer patients (80–83). Thus, the inhibition of FOXP3 expression needs further study and careful consideration regarding the role of Tregs in a given tumor microenvironment.

## Gene-Modified Tregs in Autoimmunity

Past successes using genetically enhanced T-cells in the cancer arena have prompted interest in the development of related approaches to suppress unwanted autoimmune responses. Refractory autoimmune disease is associated with a markedly decreased life expectancy urging consideration of intensive therapeutic approaches. Tregs provide an attractive tool for genetic targeting against autoantigens present in the organ(s) of interest.

### Modulating Antigen Specificity and CAR Approach in Tregs to Treat Autoimmunity

Therapeutic effect of purified Tregs have been demonstrated in preclinical studies in a range of autoimmune disease models in mice, including Systemic lupus erythematosus (84), T1D, autoimmune hepatitis, inflammatory bowel diseases, and autoimmune encephalomyelitis (85–88). Subsequently, studies in several disease model systems have demonstrated that antigen-specific Tregs were present in diseased animals and more potent in suppressing pathogenic immune responses compared to polyclonal Tregs (9, 89).

Among autoimmune diseases, T1D has been an intense area of development for gene-modified Treg-mediated therapy with islet-specific Tregs. Most recently, it has been demonstrated that lentiviral TCR gene transfer to polyclonal human Tregs achieved human islet-specific and viral-specific CD4 T cell clones. This enabled antigen-specific suppression at increased potency compared to polyclonal Tregs, increasing optimism for the success of this approach (90). However, T cells transduced with islet-specific TCRs were less responsive to cognate antigen than T cells with virus-specific TCRs, suggesting further work in this area is needed. The animal model of multiple sclerosis, EAE, has been instrumental in testing gene-modified Tregs for therapeutic intervention in neurological autoimmune diseases. For example, a lentiviral gene delivery system was used to express a CAR targeting myelin oligodendrocyte glycoprotein with the murine FOXP3 in CD4 T cells. Intranasal administration of these cells diminished ongoing neuronal inflammation *in vivo* (61).

Several other attempts to utilize CAR^+^ Tregs to treat autoimmunity have revealed the important fact that activation of Tregs needs to be antigen-specific; this was found in murine colitis and arthritis models as well as in human Treg activation (91–93). This appears a critical point since in many autoimmune disorders autoantigen(s) that trigger autoimmune responses are unknown. In the case of murine arthritis, naïve CD4^+^ T-cells were engineered to co-express FOXP3 with HLA-DR1, covalently linked to an immunodominant peptide capable of driving collagen-induced arthritis. HLA-DR1 is associated with human rheumatoid arthritis. By this approach, T-cells were equipped with a bait molecule that allowed them to engage collagen autoreactive CD4^+^ T-cells in a TCR-dependent manner. In DR1 humanized mice, the engineered T-cells could inhibit the development of autoimmune arthritis more effectively than cells engineered to express FOXP3 alone (94). However, this approach warrants further studies, among other reasons because of the distinct subset of CD4^+^CD25^+^ Tregs expressing HLA-class II in humans (95).

### Inducing FOXP3 Expression to Treat Autoimmunity

In addition to TCR modulation, modulation of FOXP3 expression itself is a promising strategy to treat autoimmune diseases. IPEX syndrome is a hereditary immunodeficiency characterized by the loss of function of FOXP3-expressing Tregs (11). A recent study demonstrated the lentiviral delivery of the FOXP3 gene into IPEX-derived CD4 T cells produced a stable Treg population. In this study, CD4 T cells from IPEX patients were converted into FOXP3-expressing Tregs, and they acquired Treg-like phenotypes *in vivo*. When FOXP3 is expressed by lentiviral gene transduction, T-cells express several Treg markers such as CD25, CTLA-4, and GITR. Functionally, the cells resembled Tregs with decreased proliferation, hypo-responsiveness, reduced cytokine release, and suppressive activity similar to purified Tregs (96). This approach for FOXP3 gene transfer with adoptive cell therapy may potentially be a promising approach to treat IPEX patients as well as other autoimmune patients with dysfunctional human Tregs. Further studies regarding the stability of FOXP3 expression in these CD4 T cells and further assessment of the efficacy of this approach in clinical settings are warranted.

Retroviral delivery of the FOXP3 gene into purified CD4^+^CD25*^−^*CD45RO*^−^* human T cells showed unstable levels of FOXP3 and Treg-associated phenotypic markers while lentiviral delivery using elongation factor-1α showed reliable expression of CD25 and GITR (97). An alternative approach may be to enforce Treg differentiation using a cell-permeable form of FOXP3 protein with a transduction domain. The introduction of FOXP3 in protein form induced a Treg phenotype in human and mouse T cells, respectively (98, 99). Repeated infusion of FOXP3 with a transduction domain showed amelioration of the *scurfy* phenotype, and inflammatory bowel disease and rheumatoid arthritis mouse models (100, 101). The cost of infusion for protein delivery in a clinical setting for human patients, the stability of a functional Foxp3 protein *in vivo*, and lack of specificity in immunosuppression due to Foxp3 protein delivery to the nucleus awaits further optimization of this approach.

## Manipulation of Tregs in Allotransplantation and Other Diseases

Clinical evaluation of adoptive immunotherapy using Tregs is attracting increasing interest. Most experience has been gained using donor-derived Tregs, which have been infused safely in patients treated with allogeneic stem cells (102). These studies have also provided encouraging evidence of efficacy in prevention of GVHD, even in the context of haploidentical stem cell transplantation (102, 103).

### Approaches to Generate Alloantigen-Specific Tregs

Similar to improved ability of autoantigen-specific Treg to control autoimmune inflammation, alloantigen-specific Tregs are more effective than polyclonal Tregs at preventing organ or tissue graft rejection. These alloantigen-specific Tregs were enriched by *in vitro* alloantigen-stimulated expansion or the expression of a TCR transgene (104–106). A humanized mouse model of skin graft rejection has also shown the potency of suppressor function of alloantigen-expanded human Tregs (107). Tregs expressing CARs could also be used in the context of transplantation. For example, a CAR approach targeting HLA-A2 has been used to produce alloantigen-specific Tregs (108). CAR-stimulated Tregs showed minimal cytotoxicity. *In vitro*, HLA-A2-CAR Tregs maintained high levels of FOXP3 expression and other Treg markers, and stable demethylation of the TSDR ensured suppressor function. The HLA-A2 approach may have significant advantages in the clinical setting where a sufficient number of APCs are required (107), and the potential loss of FOXP3 after repeated stimulation has been reported (109). With improved stability alloantigen-specific Tregs will have more versatile uses in future transplantation trials.

### Other Gene-Modification Approaches for Generating Suppressor Lymphocytes

*In vitro* generation of Tr1 cells has been developed for clinical purposes. However, a major caveat of clinical use of Tr1 cell therapy is lack of purity. Andolfi et al. showed lentiviral delivery of IL-10 and GFP could generate a homogeneous Tr1 cell population to circumvent this issue (110). These “pure” Tr1 cells showed an anergic phenotype and TGF-β/IL-10 -dependent suppression of allogeneic T-cell responses and successfully controlled GVHD (110). Tr1 cells were generated *in vitro* using genetically modified B cells in an allergy model in an antigen-specific manner. Retroviral transduction of the fusion protein, Derp 2, a major house dust mite allergen, with an endosomal targeting sequence (gp75) was performed in B cells for efficient MHC class II presentation. The engineered B cells were adoptively transferred to the host (BALB/c mice) before or after peptide immunization. The production of IL-10 from these retrogenic B cells and the induction of IL-10 expressing Tr1 cells achieved allergen-specific immune tolerance against asthma (111). Although the result is encouraging, more studies with different types of allergens, or the use of humanized mouse models, could be considered to assess the potential scope of this approach.

Corneal allograft failure is mediated by CD4 T cells (112). CD25 in CD4 T cells plays an important role in the induction of corneal graft rejection. CD25-mediated signaling is associated not only with the expression of Treg cytokines (IL-10, TGF-β) but also T helper 1 type cytokines (IFN-γ, IL-1β, and TNF-α). A recent study showed that the use of CD25 siRNA in a corneal transplantation model significantly prolonged graft survival time on Sprague-Dawley rat recipients with Wistar rat donors (113). In this study, neovascularization and maintenance of transparency of the cornea were significantly improved. However, similar studies have not been extended to human patients, and the safety of this approach remains to be tested.

## Future Perspective in Gene-Modified Treg Therapy

Recent technical advances and developments in the field of gene-modified Treg therapy have evolved into a new era. It is clear that the approach is very promising, yet several hurdles need to be overcome before broad clinical implementation. One of the biggest concerns is to ensure the purity of clinical products using GMP-based protocols. There is some concern about the stability of engineered Tregs and the fact that some Tregs might be converted into effector T cells, particularly into Th17 type cells (114, 115). Approaches that may be helpful to maintain FOXP3 expression have been discussed including all-trans-retinoic acid, IL-2, vitamin C or *ex vivo* treatment with rapamycin (116–119). For lentiviral gene transfer approaches, studies in the past showed long-term safety in human immunodeficiency virus patients who received gene-modified T-cells without genotoxic effects such as clonal expansion (120). Development in vector engineering has also achieved enhanced genetic stability and greater stability of transgene expression, providing greater safety (121). In TCR engineering, there is a concern about TCR cross-reactivity which is caused by recognition of low-affinity antigen by a TCR. There were two cases in which T cells were engrafted with an affinity-enhanced TCR selected for the tumor antigen, MAGE A3, and this TCR was found to have cross-reactivity and cardiovascular toxicity (122, 123).

## Conclusion

**Figure 1:**
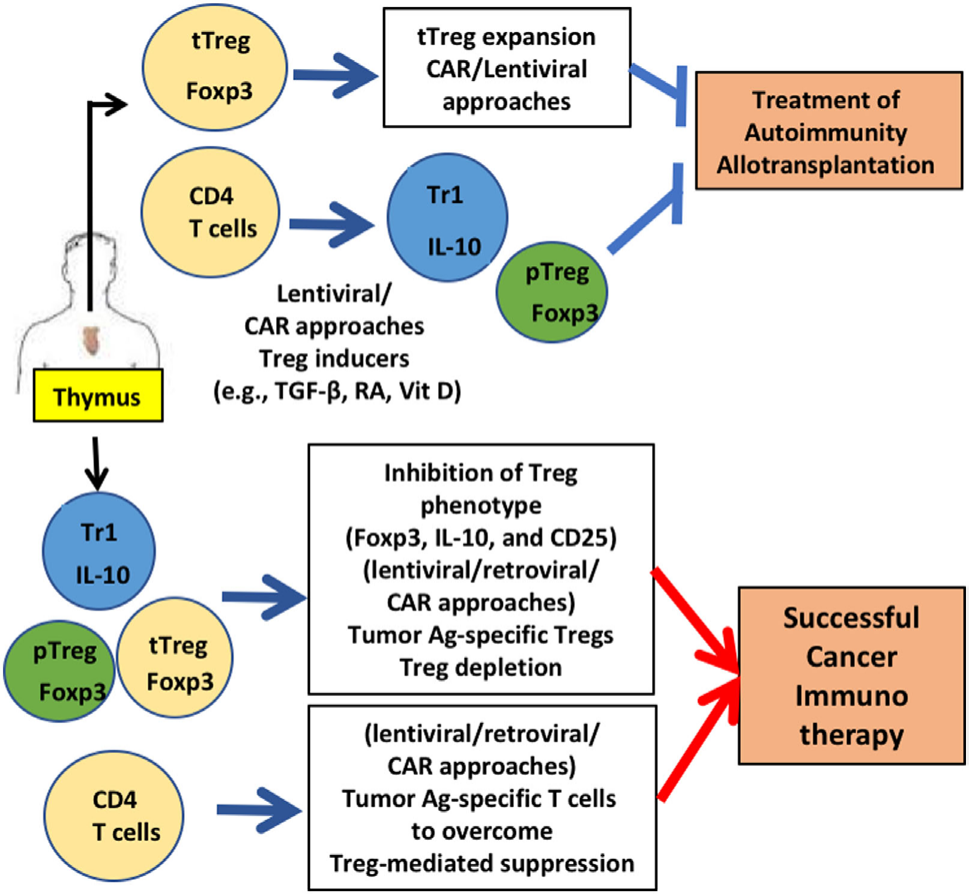
Gene-modified regulatory T cell (Treg)-mediated therapy. Engineering of Tregs aims to generate Tregs that are functionally similar to various types of Tregs that were identified in human: thymic-derived thymic-derived Treg (tTreg), transforming growth factor (TGF)-β-induced peripheral Treg (pTreg), and IL-10 expressing Tr1 cells. CD4 T cells and Tregs are generated from thymus. For treating autoimmunity or allotransplantation, gene-modification approaches aim to acquire a stable Treg phenotype and sufficient numbers *via in vitro* expansion to obtain enough cells for treatment. Generation of antigen-specific Tregs reduces the number of Tregs for therapy significantly. For successful cancer immunotherapy, Treg function needs to be downregulated. Destabilizing Treg functions by inhibiting FOXP3 and other functional Treg proteins (e.g., CD25) by gene modification is under development. Applications for these gene-modified Tregs are currently being expanded in mice (e.g., humanized mice) and men (human Tregs for clinical trials).

The regulatory functions of Tregs and specificity to various types of stimuli triggered intense research efforts to develop these cells for various clinical treatments. For example, CAR-T cells, lentiviral gene transfer, small molecule compounds that regulate FOXP3 expression, and infusion of cell-permeable FOXP3 proteins were developed (Figure 1). Potential uncertainties of gene-modified Treg therapy remain, as well as the challenges of the manipulation of Tregs under GMP conditions, and concerns of effector-mediated toxicity due to lack of purity, unstable Treg phenotypes and TCR cross-reactivity. However, alternate approaches are being sought and tested and as the clinical data emerge, these challenges shift to the further evolution of innovative therapeutic approaches.

## Author Contributions

All authors listed have made a substantial, direct, and intellectual contribution to the work and approved it for publication.

## Conflict of Interest Statement

The authors declare that the research was conducted in the absence of any commercial or financial relationships that could be construed as a potential conflict of interest.
